# Influence of the dental prosthetic status on self-perceptions of health and treatment needs: A cross-sectional study of middle-aged adults with chronic disease

**DOI:** 10.4317/jced.54783

**Published:** 2018-06-01

**Authors:** Fabiana-Barros-Marinho Maia, Emerson-Tavares de Sousa, Jossaria-Pereira de Sousa, Kelly-Guedes-Oliveira Scudine, Claudia-Helena-Soares-de Morais Freitas, Fabio-Correia Sampaio, Franklin-Delano-Soares Forte

**Affiliations:** 1 Department of Clinical and Social Odontology, Dental School, Federal University of Paraiba, Brazil; 2 Department of Pediatric Dentistry, Piracicaba Dental School, Campinas University, Piracicaba –SP/Brazil

## Abstract

**Background:**

Subjective aspects of dental prosthesis need in middle-aged adults are poorly explored, especially when the population presents chronic diseases. Objectives: To investigate if the use and necessity of dental prosthesis influence the self-perceptions of health and dental treatment need in middle-aged adults with chronic diseases.

**Material and Methods:**

A cross-sectional study was performed in 210 middle-aged adults. Socio-demographic aspects, dental services use, oral and general perceptions of health, dental treatment need and OIDP were assessed using a standardized questionnaire. One trained dentist evaluated the use and necessity of dental prosthesis. Data were analyzed using Chi-square and multivariate logistic regression (*p*<0.05).

**Results:**

The use of dental prosthesis showed a proportion of 2.2:1 for upper to lower arch. Dental prosthesis need was largely prevalent (90 and 95% in upper and lower arch, respectively). The self-perceptions of dental treatment need and oral health were associated with the use and necessity of dental prosthesis (*p*<0.05), which could not be observed in relation to the general self-perception. The adjusted model demonstrated that the subjective necessity of dental treatment was 16.04 (1.92-133.7) fold higher in individuals with necessity of rehabilitation in the lower arch. Besides, a positive self-perception of oral health (satisfied) was 2.59 (1.38-4.85) more expressive in individuals that used a dental prosthesis in upper arch.

**Conclusions:**

The self-perception of oral health was influenced by the use of maxillary dental prosthesis in individuals with chronic disease. Moreover, individuals were more likely able to perceive treatment need when the lower jaw was affected by tooth loss.

** Key words:**Tooth loss, epidemiology, personal satisfaction, aged, chronic disease.

## Introduction

Despite the global trend of tooth loss reduction over the last decades, the edentulism is still high in many parts of the world (1). In this context, tooth loss is a public health problem closely associated with morphofunctional and psychosocial changes (2,3). Besides, from a public health point of view, the edentulism can be considered a social marker of oral diseases experienced over the time, reflecting a legacy of an assistance model based on mutilating practices (4).

From an epidemiological perspective, individuals older than 50 years present more severe levels of tooth loss. This fact has political implications since it evidences the need for integral actions in oral health, which should include the multidimensional construct that reflects all aspects of the aging process (5). In light of this, the integral understanding of individuals in this age group (middle-aged and elderly individuals) is important to expand the subjective concepts of general and oral health. Moreover, the separation of the mouth from the body is a cultural context that promotes a particular impact on health care attitudes and the motivations for seeking treatment (6).

Recently, it has been demonstrated that the tooth loss is associated with chronic diseases such as hypertension and diabetes (7,8). However, it is difficult to understand the pathophysiological mechanisms underlying these associations due to the absence of evidence regarding causality. In fact, it has been demonstrated that physically, medically or pharmaceutically affected individuals have worse oral health status, which can affect functionally and socially the people’s daily life (9). In this regard, it was well reported that common risk factors (e.g. sugar intake, smoking, alcoholism, poor education, low socioeconomic status) possibly promote a higher risk of oral and systemic multimorbidity (10).

Since the broad comprehension of patients during each phase of life is needed to monitor and control risk factors of illness, the investigation of personal perception of health should be considered as an essential part of the caring in public health (11). This perspective also includes an integral caring of individuals into a social, cultural, and historical context. Moreover, considering the tooth replacement as an elective treatment, the investigation of subjective factors involved in patient perceptions of health and the reasons that possibly motivate seeking for treatment are of particular importance.

The use of patient-centered questionnaires addressed to self-rated oral health offers advantages in the planning and management of public oral health services, providing a reliable view of the patients’ demands (12,13). In this framework, the self-perceptions of general and oral health, as well as the self-concept of treatment need, has been described as an important predictor of treatment seeking, and a reflex of individuals’ attitudes in relation to their own oral conditions (14). However, considering the subjective nature of these analyses, the necessity of prosthetic rehabilitation is a controversial field.

The extensive tooth loss is responsible for physiologic and psychosocial complications (2,3); nonetheless, the way of people experience this condition is widely diverse. The scientific literature has demonstrated that subjective indicators could act as predictor factors of individuals’ prosthetic status (14); however, there is no consistent information about the subjective aspects on the necessity of dental prosthesis in a representative sample of middle-adults with chronic conditions. Thus, this field study investigated if the use and necessity of dental prosthesis influence the self-perceptions of health, treatment need and Oral Impact on Daily Performance (OIDP) in middle-aged adults with chronic disease.

## Material and Methods

The present study adheres to the STROBE statement for reporting of cross-sectional studies.

-Ethical Considerations 

This study was approved by the research ethics committee of Federal University of Paraiba (CAAE: 35493114.4.0000.5188) and was conducted in accordance with all principles of Helsinki Declaration, respecting the participant’s consent formalized by the signature in a consent form.

-Study area 

The target sample was recruited by an industrialized and medium-sized city (726 km²) in northeastern of Brazil (Santa Rita/PB/Brazil). Data from the “Human Development Atlas” provided by the Brazilian agency of United Nations Development Program (http://www.pnud.org.br) shows that the city presents a Human Development Index (HDI) of 0.63 and a Gini index of 0.46. The healthcare in this region was conducted according to the Unified Health System (SUS in Portuguese) politics that promotes preventive and therapeutic interventions by a network of 42 Family Health Care Strategy (FHCS). The oral health care is present in primary attention by the integration of a general dentist for each 42 family health teams, to provide access to 83.81% of the population (http://www.datasus.gov.br).

Considering the integrality of the health care in Brazilian public system, there are many programs of health monitoring. Thus, following the purpose of this paper, we focused on two: the ‘Hiperdia’ program (15) and Smiling Brazil (16). The first one is an operational program for management of non-communicable disease in public health, and the latter is a public politician designed to promote actions for oral health care.

-Study Design and Sample 

A cross-sectional study was performed involving a random sample of adults between 50-65 years old that have hypertension and/or diabetes. The research was designed considering two phases. First, the selected individuals were analyzed by a calibrated dentist that collected information about DMTF and the dental prosthetic status (use and necessity of dental prosthesis). Second, the volunteers were advised to answer a structured interview about sociodemographic aspects (gender, schooling, and income), use of dental care services (last visit and type of service) and self-perception of the necessity of prosthetic treatment, general and oral health.

The estimated sample size was 210 individuals, assuming the population proportion of 0.70 of dental prosthesis need (17), alpha of 0.05 for the two-sided confidence level, Estimated Design Effect (DEFF) of 1, the desired level of absolute precision of 0.06, and 95% of significance. The sampling method (two-stage conglomerate) was carried out in SPSS software that performs the lottery. In the first stage, one FHCS was selected at random of the 42 FHCS enrolled in Basic Attention Information System.

The second stage considered the aleatory selection of individuals from ‘Hiperdia’ Program and inclusion of the sample according to the eligibility criteria. The eligibility criteria were individuals 1) with medical diagnosis of type 2 diabetes and/or hypertension, 2) aged between 50-65 years old, 3) literate, 4) signed the consent form, 5) filled out completely a questionnaire with no items missed, and 6) do not present any physical or/and mental impediment to answering the interview questions.

-Pilot Study 

Before the beginning of the research, the examiner (Maia, FBM) and a dental assistant were trained by an oral epidemiologist (Forte, FDS), in a sample with similar age. The training exercise was performed with a theoretical and clinical phase. The theoretical phase involved the analysis of WHO guidelines and photographs cases following a discussion regarding the use of dental prosthesis and the necessity of use (18). The clinical phase was executed with 30 randomly selected participants who were not included in the main sample. The reliability was checked twice within a 2-week period and assessed by kappa test (k= 0.98). Results of the pilot study also revealed no misunderstandings regarding the questionnaire methodology and comprehension.

-Clinical Collection

Only one examiner performed the clinical examination at volunteer’s home, in a face-to-face position in the presence of natural light. A sterilized mouth mirror and a WHO probe (Golgran Ind. E Com. Ltda., São Paulo, SP, Brazil) were used to visual inspection according to the WHO recommendations for epidemiological oral health surveys (18). The examiner used the personal protective equipment.

The use and necessity of dental prosthesis were measured in the upper and lower arch (18). Both variables represented dependent variables as is described below:

a) Use of dental prosthesis: Considered the use of total, partial and fixed prosthesis, according to the presence of a prosthetic space (tooth loss).

b) Necessity of dental prosthesis: Considered the presence of prosthetic space without rehabilitation or the use of a dental prosthesis with low quality. The quality of dentures was evaluated with respect to clinical parameters such as retention (loose or tight), stability (presents displacement or scale), aesthetics (stains or fractures), and fixation (promotes lesion on oral tissues). If at least one of these conditions is present, the individual was classified as positive to the normative necessity of prosthesis replacement.

-Non-clinical Collection 

A questionnaire was conducted with objective questions addressed to independent variables like socioeconomic characteristics, use of dental services, self-perception of general and oral health, and self-perception of dental treatment need. Additionally, to investigate the recent impact of oral health conditions on daily performance, the OIDP was applied (19). The item “emotional stability” was removed due to problems with translation and possible misinterpretation. The questions and options of replies are available in Box 1.

-Statistical Approach

Statistical analysis was performed using SPSS software 21.0 (SPSS, Inc., Chicago, IL, USA). The Chi-square (χ2) and Fisher’s exact tests were performed to test the association between variables. The significance level was set at *p* < 0.05 (two-tailed). The multivariate logistic regression assessed the relative influence of the independent variables and the use/necessity of dental prosthesis. The odds ratio (OR) and p-value were calculated to perform a model of explanation considering the biological plausibility of the primary outcome in upper and lower arch. This statistical procedure was performed based on stepwise regression through backward elimination, and represented the adjusted covariables, with predictive value significant when *p*≤0.05.

## Results

The average DMFT was 27.76 (± 5.62), with a predominance of the “lost” component (22.92 ± 8.61). The edentulism was present in 32% of the sample. Results of  Table 1 demonstrated that the use of dental prosthesis is highly common in upper arch than lower arch, following a proportion of 2.2:1. Similarly, it was noticed that the necessity of dental prosthesis is largely prevalent considering the absence of prosthesis (no use) and the low quality of these devices (necessity of changing the prosthesis).

**Table 1:**
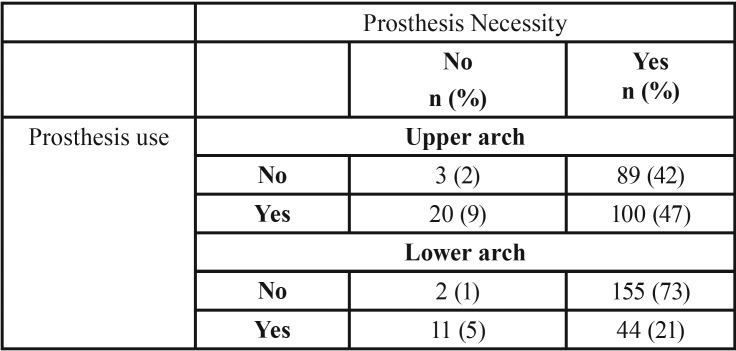
Use and necessity of dental prosthesis in patients with chronic disease.

In the bivariate analysis, the self-perceptions of dental treatment need and oral health were associated with the use and necessity of dental prosthesis (*p*<0.05), however, this trend was not found regarding the general self-perception ( Table 2).

**Table 2:**
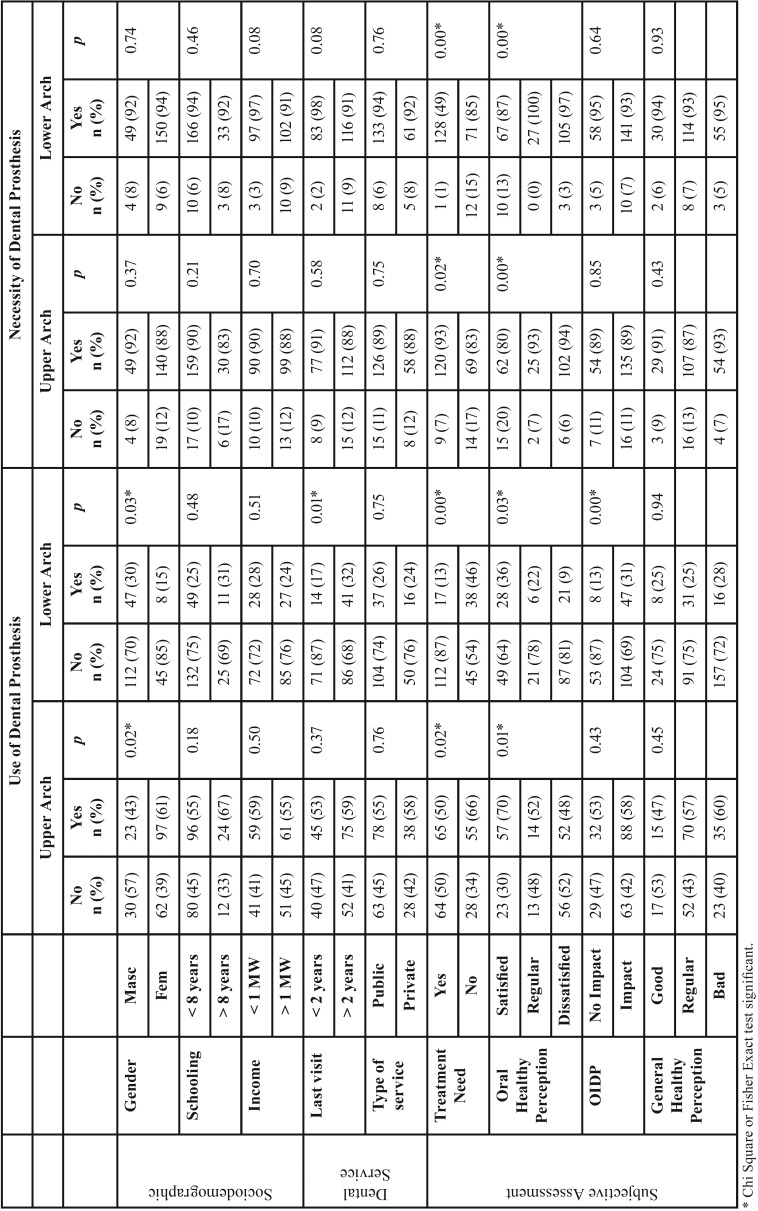
Factors associated with the use and necessity of dental prosthesis.

In  Table 3, it was observed that the self-perception of treatment need was 16.04 fold higher in individuals with the necessity of rehabilitation in the lower arch. The positive self-perception of oral health (satisfied) was 2.59 more expressive in individuals that used a dental prosthesis in upper arch. This penchant can be detected in relation to the objective necessity of dental prosthesis, which indicates that the satisfaction with oral health is 75% smaller in individuals with the need of upper prosthetic devices.

**Table 3:**
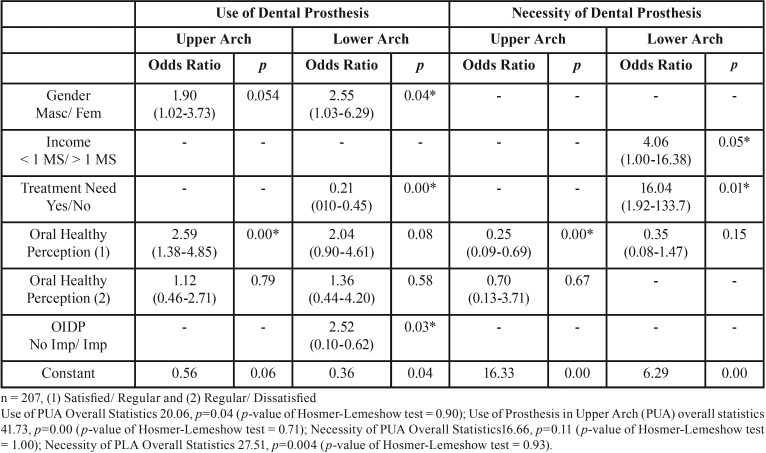
Multiple logistic regression of the use and necessity of dental prosthetic.

## Discussion

Results of this cross-sectional research evidenced that the use and necessity of dental prosthesis were associated with subjective perceptions of oral health and treatment needs. Moreover, our results suggest that the self-perceptions of health and treatment need, and OIDP can be modified according to the dental arch replacement. These outcomes provide the better understanding regarding individuals’ self-perception of health as well as clarify aspects related to individuals’ expectations and seek for treatment.

Similar to a previous Brazilian study (13), tooth loss was prevalent in both upper and lower jaw. Part of these results can be explained by the curative model predominant in public oral health care system in Brazil before the implementation of Unified Health System (SUS) in 1989, which was limited to serial extractions and emergency services (20). Brazil’s National Oral Health Policy was established with the implementation of Smiling Brazil program in 2004, which have made access to dental care easier. The philosophy of Smiling Brazil program is centered on the individual care and the contextual prevention of oral diseases (16). However, in a pragmatic point of view, the biomedical health care model still prevails and the Smiling Brazil program has not yet achieved a decrease in the existing socioeconomic inequalities in dental health service use (21).

In a biological perspective, the high prevalence of tooth loss can be justified by the presence of chronic disease. This statement was deeply studied by Singh *et al.* (8) and Gil-Montoya *et al.* (22) who found a significant association between tooth loss and hypertension and diabetes, respectively. The loss of a functional dentition may affect the dietary quality and nutrient intake, resulting in obesity and high blood pressure (23,24). Regarding diabetes, one potential pathway is oral infection-inflammation related to periodontal disease (22).

Dental prosthesis use in the upper arch was higher than in the lower arch. This finding did not necessarily indicate a non-necessity of upper prosthesis since half of the individuals needed prosthesis in the maxilla presented an unsatisfactory unit in the same arch. In contrast, the need for prosthesis on lower arch was more prevalent in subjects who did not use any prosthesis. The combination of these results highlights problems surrounding public health (social inequality), indicating the necessity of greater efforts and maybe a different strategy to reduce mutilating practices among populations and exposes a suppressed demand for prosthetic rehabilitation.

In Brazil, the public oral health care was designed to provide a universal and full care to the population. This process started with the creation of Unified Health System in 1988 and was established with the implementation of Smiling Brazil program in 2004, which have made access to dental care easier. In this sense, considering the policy basis of Brazil’s National Oral Health, it is emphasized the importance of the primary and secondary health care as a potential space for innovations and changes in the oral health work process.

The high prevalence of total edentulism (32%) and a remarkably suppressed demand for prosthetic rehabilitation among middle-aged adults with chronic disease reproduces a reality of oral and systemically affected individuals. High rates of tooth loss in individuals with chronic conditions were found in several studies (8,25). The accumulated demand can be a consequence of factors as behavior, lifestyle, oral health politics, health service availability, and socioeconomic structure (26). In this context, it is necessary to reemphasize the importance of interdisciplinary and interprofessional integration to promote an integral caring in health services, avoiding permanent oral complications in vulnerable populations.

The multiple logistic regression revealed that subjective issues could be associated with the normative assessments of use/necessity of dental prosthesis depending on the arch evaluated. Individuals with good self-perception of oral health had 2.5 more chance to use a maxillary prosthesis. Besides, a decrease of 75% in the maxillary dental prosthesis necessity was observed in individuals with a good perception of oral health. It indicates that the replacement of the upper arch can be considered more aesthetically suitable than the lower arch. Upper dental prothesis use can decrease the adverse effects of tooth loss by improving self-esteem and interpersonal relationships (27). There are several factors that influence the people’ aesthetic perceptions such as ethnicity, race, gender, age, and facial anatomy (28). However, a proper understanding of what is considered esthetic for the patients’ point of view cannot be answered by this study.

The self-perception of treatment need reflects the impact of diseases on individuals’ life by evidencing the degree of deficiencies and dysfunctions experienced, as well as the individuals’ perceptions and attitudes regarding those conditions (29). Besides, the subjective necessity as a complement of the normative necessity can be considered a possible epidemiological tool for planning oral health care services (14). The multilevel logistic regression identified a strong association between the self-perception of treatment need and the normative necessity of lower denture, showing that subjects who report necessity of dental treatment had 16.04 more chance of needing rehabilitation in the lower arch. As a controversy, the necessity of lower dental prosthesis is negligible for the patients’ self-perceptions of oral health. The most likely explanation is that the concept of dynamic occlusion and masticatory function are not completely understood in the patient’s reality (29). In other words, irrespective of the contextual situation, individuals know that the replacement of the lower arch is necessary but they do not think that it is important.

OIDP index is a psychometric scale intrinsically associated with the oral health-related quality of life (19). In the current investigation, it was verified that the absence of dental prosthesis in the lower jaw is a risk factor (odds ratio: 2.52) to the OIDP impact. A recent report observed similar results in a Brazilian population of adults, demonstrating that subjects dentate and wearing lower denture presented a reduced impact on oral health-related quality of life (12). These results suggest that the absence of a functional antagonist arch may play an important role in the oral impact on daily performance in individuals. Possible explanations that could be outlined is the loss of confidence, the limitation of food choice, the reduction in the enjoyment of food, the avoidance of laughing in public, and the reluctance to form close relationships (30).

Results of this study provide a pioneer and important contribution because expands the understanding of individual and community aspects of illness in middle-aged and chronic affected individuals. Moreover, the present study was carried out with a proper calculation of the sample size and use of the multilevel statistical technique. Having said that, we have pointed out some limitations as the cross-sectional design (did not allow us to evaluate causal relationships), the absence of difference among the type of prosthesis used or needed (implant-supported, fixed, total or partial removable prosthesis), and the absence of control group (without chronic disease).

## Conclusions

Dental prosthesis use in upper arch influences the self-perceptions of oral health, as well as the necessity of mandibular dental prosthesis influences the self-concept of treatment need in individuals that experienced both tooth loss and non-communicable disease.
